# Intravoxel incoherent motion combined with conventional MRI for the differentiation of benign, intermediate, and malignant fibrous soft-tissue tumors

**DOI:** 10.3389/fonc.2026.1863609

**Published:** 2026-06-26

**Authors:** Yue Dai, Yuhan Long, Jie Zhou, Juan Tao, Wenjia Wang, Yifeng Zhu, Kai Zhang

**Affiliations:** 1 Department of Radiology, The Second Hospital of Dalian Medical University, Dalian, China; 2 Department of Radiology, Central Hospital of Dalian University of Technology, Dalian, China; 3 Department of Pathology, The Second Hospital of Dalian Medical University, Dalian, China; 4 MR Research Center China, GE Healthcare China Co Ltd, Beijing, China

**Keywords:** diffusion-weighted imaging, fibrous soft-tissue tumors, intravoxel incoherent motion, magnetic resonance imaging, qualitative diagnosis

## Abstract

**Background:**

Fibroblastic/myofibroblastic tumors are among the most common soft-tissue tumors (STTs) encountered clinically. Several magnetic resonance imaging (MRI) features associated with malignant tumors overlap with benign tumors, making differential diagnosis challenging. Intravoxel incoherent motion (IVIM) is a valuable MRI technique for differentiating various tumors. This study aims to evaluate the abilities of conventional MRI and IVIM in differentiating benign, intermediate, and malignant fibrous STTs.

**Methods:**

Fifty-five patients with fibrous STTs were prospectively enrolled, comprising 18 benign, 18 intermediate, and 19 malignant cases. All the patients underwent MRI examinations including IVIM. Conventional MRI signs and standard-apparent diffusion coefficient (ADC), true diffusion coefficient (D), pseudo-diffusion coefficient (D^*^), and perfusion fraction (f) were recorded. Statistical analyses were performed using Kruskal–Wallis H test, Chi-square test,*post hoc* test with Bonferroni correction, receiver operating characteristic (ROC) curves, and DeLong test. p < 0.05 indicated statistical significance.

**Results:**

Malignant tumors had higher heterogeneity on T2WI (*p* = 0.020 and 0.009) and contrast enhancement T1WI (*p* = 0.013 and 0.029), and were more prone to necrosis (*p* < 0.001 and*p* = 0.001) compared with benign and intermediate tumors, respectively. Tail-like pattern (*p* = 0.034 and 0.009) and invasiveness (*p* = 0.018 and 0.033) were more frequently observed in intermediate and malignant tumors than in benign tumors, respectively. Standard-ADC_mean_, standard-ADC_min_, D_mean_, and D_min_ values decreased from benign to intermediate and malignant fibrous STTs. Malignant STTs displayed higher f_mean_ and f_min_ values than benign tumors (*p* = 0.002 and 0.013, respectively). Standard-ADC_mean_ showed the highest AUC (0.894) in differentiating intermediate from benign STTs. D_mean_ showed the highest AUC (0.961 and 0.905) in differentiating malignancies from benign and intermediate STTs, respectively. For discriminating between benign and non-benign fibrous STTs, the combination of conventional MRI signs and IVIM parameters yielded the highest AUC of 0.971.

**Conclusion:**

IVIM diffusion parameters differentiated benign, intermediate, and malignant fibrous STTs and can complement conventional MRI signs.

## Introduction

Fibroblastic and myofibroblastic tumors, or fibrosis tumors, are a subtype of soft tissue tumors (STTs) in the 2020 World Health Organization (WHO) classification of STTs (1). Fibrous tumors are one of the most common subtypes of STTs encountered clinically, with three subcategories: benign, intermediate, and malignant. Benign fibroblastic tumors, such as nodular fasciitis, have a self-limited course and can be followed up with observation (2). Intermediate tumors, including solitary fibrous tumors (SFTs), typically require extensive surgical resection; a watch-and-wait strategy is required for desmoid-type fibromatosis (DF), and advanced lesions should be treated pharmacologically (3). Malignant fibrosarcomas require surgery combined with adjuvant chemoradiotherapy or immunotherapy (4,5). For all fibroblastic/myofibroblastic tumors, accurate early diagnosis and timely intervention are essential for improving patient prognosis.

Magnetic resonance imaging (MRI), which integrates multiple signal and morphological characteristics, is the preferred method for diagnosing and evaluating STTs. Conventional MRI findings suggestive of fibrous tumors include hypointense areas on T1-weighted image (T1WI) and T2-weighted image (T2WI) formed by fibrous stroma, irregular shape, and linear extension along fascial planes (6–10). Specific MRI features such as larger size, deep location, high heterogeneity, necrosis, and peritumoral edema have been reported to be associated with fibrosarcomas (11). However, some benign tumors, previously known as “pseudosarcomas,” which are characterized by rapid growth, hypercellularity, and infiltrative ability, such as nodular fasciitis and proliferative myositis, may also exhibit these MRI features (2,6). The overlapping of these histological and radiological manifestations makes accurate differential diagnosis challenging.

Diffusion-weighted imaging (DWI) has improved the clinical utility of MRI by offering insights into tissue cellularity, and these findings have supported the differential diagnosis of STTs (12–15). However, the apparent diffusion coefficient (ADC) values derived from the monoexponential DWI model not only reflect the diffusion of water molecules but are also influenced by microvascular perfusion, which can influence the diagnosis of benign or malignant lesions. Thus, relying solely on DWI for characterizing STTs remains limited. Intravoxel incoherent motion (IVIM) is a technique that separates molecular diffusion from capillary perfusion within each voxel and yields the true diffusion coefficient D along with capillary perfusion–related parameters: the pseudo-diffusion coefficient D^*^ and perfusion fraction f (16). These parameters are widely used in the differential diagnosis of diverse tumors. Studies evaluating the ability of the biexponential IVIM model to distinguish benign STTs from malignant STTs have yielded inconsistent results (17–20). Few investigations using IVIM have focused specifically on fibrous tumor subtypes.

In this study, we evaluated the clinical utility of IVIM combined with conventional MRI for the preoperative differentiation of benign, intermediate, and malignant fibrous STTs in the limbs and trunk.

## Materials and methods

### Patients

**Figure 1:**
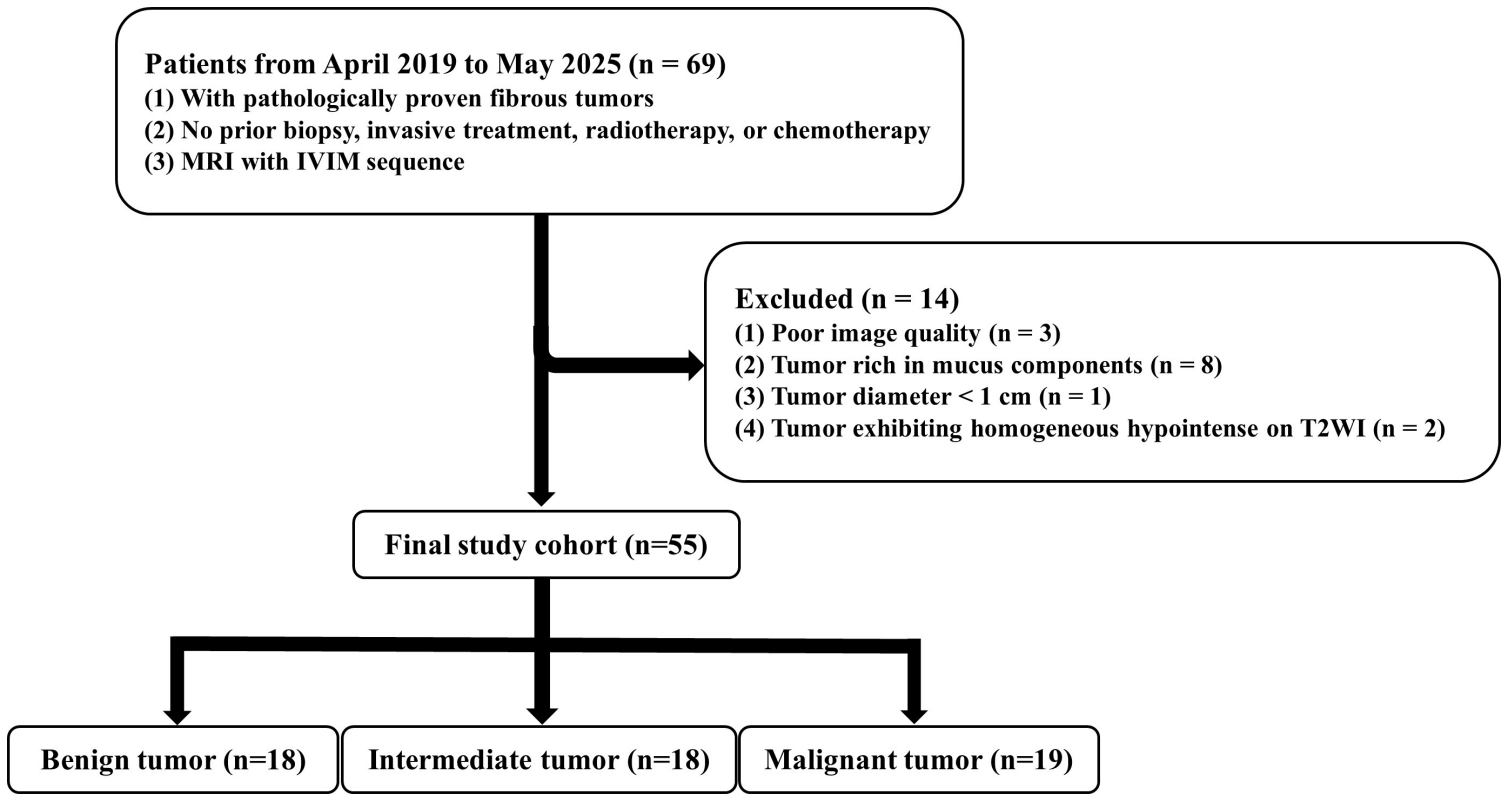
Study flowchart.

The Hospital Ethics Committee approved the design of this prospective study, and informed consent was obtained from all participants. The inclusion criteria were as follows: 1) patients with pathologically proven fibrous tumors; 2) no prior biopsy, invasive treatment, or chemoradiotherapy before initial MRI; and 3) IVIM sequence acquisition on a specific scanner (Discovery MR750w, GE Healthcare). Exclusion criteria were as follows: 1) poor image quality with severe artifacts or lesion distortion(n = 3); 2) tumor rich in myxoid components (n = 8); 3) tumor diameter < 1 cm (n = 1); and 4) tumor exhibiting homogeneous hypointense on T2WI (n = 2) (**Figure 1**). Finally, 55 patients with fibroblastic/myofibroblastic tumors were included in this study. Histopathological examination served as the gold standard reference for assessment in this study. The tumors were classified as benign, intermediate, or malignant following the 2020 WHO classification of STTs.

### MRI examination

MR images were acquired with the patient in a supine position. Coils (surface, knee, or body coil) were used depending on the anatomical region being imaged. Conventional MRI sequences included T1WI (repetition time [TR]/echo time [TE], 478–689/13–19 ms; echo train length [ETL], 3; slice thickness, 3 mm; slice gap, 1 mm; number of excitations [NEX], 2) and T2WI (TR/TE, 2900–5521/70–96 ms; ETL, 19; slice thickness, 3 mm; slice gap, 1 mm; NEX, 2) fast spin-echo (FSE) sequences, performed with or without fat suppression. The matrix and field of view (FOV) were adjusted on the basis of tumor size. The sequences were acquired primarily in the axial plane, supplemented by sagittal or coronal planes depending on the location and morphology of the lesion. IVIM was performed using spin-echo echo-planar imaging (SE-EPI) sequences at the axial position prior to contrast medium injection. The slice thickness, slice gap, and FOV for SE-EPI matched those of the conventional MRI sequences. Eight b values (0, 25, 50, 75, 100, 200, 500, 800 s/mm^2^) were used, with NEX, 2–4; TR/TE = 3000/70 ms; and scan time, 153 s.

### MRI analysis

#### Quantitative analysis

Raw IVIM data were processed on an Advantage Workstation (ADW 4.7, GE Healthcare). Multi-b-value DWI images were analyzed using Functool-MADC software, and background noise was reduced by adjusting the intensity threshold. A monoexponential model was applied to generate the standard-ADC map using b-values of 0 and 800 s/mm^2^. A biexponential model yielded parametric maps for D, D^*^, and f. D represents the true diffusion coefficient, reflecting the diffusion of water molecules outside the microcirculation. D^*^ denotes the pseudo-diffusion coefficient, associated with microcirculatory perfusion. f corresponds to the perfusion fraction, indicating the proportion of perfusion effect within the total diffusion effect.

Two radiologists with 8 and 18 years of experience, blinded to clinicopathological data, placed regions of interest (ROIs) on the ADC maps using conventional MRI images for spatial reference. ROIs included the largest solid portion of each tumor; areas of cystic changes, necrosis, hemorrhage, and edema, tumor margins, and gross artifacts were avoided. The mean and minimum values of standard-ADC, D, D^*^, and f in each ROI were recorded. The average of the measurements from the radiologists was calculated and recorded.

#### Qualitative MRI analysis

The two radiologists independently recorded conventional MRI signs for each tumor: 1) tumor size, defined as the longest measurable diameter of the tumor; 2) Rald, defined as the ratio of axial and lateral diameter, calculated using the formula Rald = dorth/dmax, where dmax represents the maximum tumor length and dorth denotes the corresponding maximum tumor width in any perpendicular orientation; 3) T1WI and T2WI predominant signal intensity (SI), defined as the predominant SI of the solid component relative to the skeletal muscle, categorized as iso-/hypointense or hyperintense; (4) heterogeneous SI on T1WI, T2WI, and contrast enhancement T1WI, defined as the percentage of low, intermediate, and high mixed SI in the tumor, categorized as ≥30% heterogeneity regions or <30% heterogeneity regions; (5) tissue layer, defined as the depth of the tumor relative to the subcutaneous fat and superficial fascia, categorized as deep or superficial; (6) margin, defined as the relationship between the tumor and adjacent surrounding structures, categorized as poorly- or well-defined; (7) shape, categorized as lobulated or oval; (8) band sign, defined as a band-like linear hypointense on T2WI, categorized as present or absent; (9) necrosis, defined as significant hyperintense on T2WI without enhancement after injection of contrast medium, categorized as present or absent; (10) flow void, defined as tubular and branching vascular hypointense on T2WI, categorized as present or absent; (11) tail-like pattern, defined as linear extension along fascial or skin planes with enhancement after injection of contrast medium, categorized as present or absent; (12) invasiveness, defined as the presence of one or more of the following signs: growth of the mass across the muscle fascia, invasion into the surrounding fat space, interruption or entrapment of peripheral nerves and blood vessels, or destruction of adjacent bone; categorized as present or absent; (13) peritumoral edema, defined as fluid-like hyperintense around the tumor on fat suppression T2WI that is clearly distinguished from the tumor entity, categorized as present or absent; and (14) peritumoral enhancement, defined as contrast enhancement on T1WI after gadolinium chelate injection beyond the apparent tumor border, categorized as present or absent.

### Statistical analysis

Statistical analyses were performed with SPSS ver. 25.0 and MedCalc ver. 15.8. The Kolmogorov–Smirnov and Levene tests assessed normality and homogeneity of variance, respectively. Normally distributed data are reported as mean ± standard deviation, while non-normally distributed data are expressed as median (interquartile range). Quantitative and qualitative parameters of the three fibrous STT types were compared using the Kruskal–Wallis H test and the chi-square test, respectively.*Post hoc* pairwise comparisons used Dunn’s test with Bonferroni correction. Interobserver agreement for qualitative variables was evaluated using weighted kappa (κ) statistics, interpreted as follows: <0, poor; 0–0.20, slight; 0.21–0.40, fair; 0.41–0.60, moderate; 0.61–0.80, substantial; and 0.81–1.00, almost perfect. For quantitative parameters, intraclass correlation coefficients (ICCs) were assessed; values exceeding 0.75 indicated good agreement. The area under the receiver operating characteristic curve (AUC) was calculated to assess the diagnostic performance of quantitative parameters, while optimal cutoff values, sensitivity, specificity, and accuracy were determined using the Youden index. Logistic regression analysis was applied to evaluate the combined diagnostic performance of multiple parameters, and the DeLong test compared the resulting AUCs. A p-value less than 0.05 indicated statistical significance.

## Results

### Study population

The study included 55 patients (45.5% male; 54.5% female) with fibrous STTs, with a mean age of 55 years (range, 23–81 years). The 55 STTs included 18 benign STTs, 18 intermediate STTs, and 19 malignant STTs. The most frequently involved anatomical location was the trunk (n = 15), followed by the upper limb (n = 12), thigh (n = 9), shoulder (n = 7), distal lower limb (n = 6), hip (n = 6). The malignant degree of tumors was not related to sex and location, but increased with age, which is in line with the epidemiological features of STTs (**Table 1**).

**Table 1:** Demographic data and tumor characteristics.

Clinical features	Benign (n = 18)	Intermediate (n = 18)	Malignant (n = 19)	P value
Age (years)	43.06 ± 16.97	46.28 ± 15.51	67.05 ± 15.62	<0.001
Sex				0.182
Female	8/18 (44.4)	13/18 (72.2)	9/19 (47.4)	
Male	10/18 (55.6)	5/18 (27.8)	10/19 (52.6)	
Location				0.324
Limb	15/18 (83.3)	11/18 (61.1)	14/19 (73.7)	
Trunk	3/18 (16.7)	7/18 (38.9)	5/19 (26.3)	
Histological diagnosis	Nodular fasciitis (n = 8)	Dermatofibrosarcoma protuberans (n = 7)	Fibrosarcoma (n = 15)	
	Proliferative myositis (n = 1)	Desmoid-type fibromatosis (n = 8)	Myofibroblastic sarcoma (n = 1)	
	Myositis ossificans (n = 1)	Inflammatory myofibroblastic tumor (n = 2)	FS-DFSP (n = 3)	
	Fibroma of tendon sheath (n = 2)	Solitary fibrous tumor (n = 1)		
	Elastofibroma (n = 2)			
	Angiofibroma (n = 2)			
	Acral fibromyxoma (n = 1) Desmoplastic fibroblastoma (n = 1)			

FS-DFSP, fibrosarcomatous dermatofibrosarcoma protuberans.

### Inter-examiner agreement

For the qualitative MRI features, the interobserver agreement showed substantial to almost perfect agreement (κ = 0.604–0.890) (**Table 2**). For the quantitative parameters, good inter-examiner agreement was observed (ICCs: 0.780–0.981), except for R_ald_ (ICC = 0.673), f_mean_ (ICC = 0.654), and f_min_ (ICC = 0.569) (**Table 3**). ICC analyses were further implemented for all quantitative parameters stratified by anatomical site, with results summarized in **Supplementary Table 1**.

**Table 2:** Inter-examiner agreement for the measurements of qualitative MRI features.

Parameters	kappa coefficient	95%CI
T1WI predominant SI	0.849	0.706–0.992
T2WI predominant SI	0.780	0.586–0.974
Heterogeneous SI on T1WI	0.604	0.365–0.843
Heterogeneous SI on T2WI	0.700	0.498–0.902
Heterogeneous SI on contrast enhancement T1WI	0.817	0.645–0.989
Tissue layer	0.890	0.768–0.999
Margin	0.741	0.563–0.919
Shape	0.701	0.511–0.891
Band sign	0.824	0.634–0.999
Necrosis	0.676	0.458–0.894
Flow void	0.698	0.473–0.923
Tail-like pattern	0.657	0.424–0.890
Invasiveness	0.722	0.530–0.914
Peritumoral edema	0.638	0.438–0.837
Peritumoral enhancement	0.855	0.718–0.992

CI, confidence interval; T1WI, T1-weighted image; SI, signal intensity; T2WI, T2-weighted image.

**Table 3:** Inter-examiner agreement for the measurements of quantitative parameters.

Parameters	ICC	95%CI
Tumor size	0.981	0.968–0.989
Rald	0.673	0.498–0.795
Standard-ADCmean (×10–3 mm2/s)	0.885	0.810–0.931
Standard-ADCmin (×10–3 mm2/s)	0.780	0.650–0.865
Dmean (×10–3 mm2/s)	0.928	0.879–0.957
Dmin (×10–3 mm2/s)	0.891	0.821–0.935
D*mean (×10–3 mm2/s)	0.815	0.702–0.888
D*min (×10–3 mm2/s)	0.788	0.662–0.871
fmean	0.654	0.471–0.782
fmin	0.569	0.360–0.724

ICC, Intraclass correlation coefficient; CI, confidence interval; R_ald_, ratio of axial and lateral diameter; ADC, apparent diffusion coefficient; D, true diffusion coefficient; D^*^, pseudo-diffusion coefficient; f, perfusion fraction.

### Conventional MRI findings

The examined conventional MRI features are summarized in **Table 4**. We found that tumor size, heterogeneous SI on T2WI, heterogeneous SI on contrast enhancement T1WI, necrosis, tail-like pattern, and invasiveness all significantly differed among benign, intermediate, and malignant fibrous STTs (all*p* < 0.05). Pairwise comparisons revealed that compared with benign STTs, malignant STTs were significantly larger in size (mean, 6.21*vs*. 4.31 cm;*p* = 0.040), with higher heterogeneity on T2WI (*p* = 0.020) and contrast enhancement T1WI (*p* = 0.013), and were more prone to necrosis (*p* < 0.001), tail-like pattern (*p* = 0.009), and invasiveness (*p* = 0.033). Malignant tumors had higher heterogeneity on T2WI (*p =* 0.009) and contrast enhancement T1WI (*p =* 0.029) compared with intermediate tumors and were more prone to necrosis (*p =* 0.001). Tail-like pattern (*p* = 0.034) and invasiveness (*p* = 0.018) were more frequently observed in intermediate tumors than benign tumors. We combined intermediate and malignant tumors into the non-benign group, and further analysis revealed that these tumors were significantly larger than benign tumors (mean, 5.83*vs*. 4.31 cm;*p* = 0.023) and more frequently associated with necrosis (*p* = 0.005), tail-like pattern (*p* = 0.004), and invasiveness (*p* = 0.008).

**Table 4:** Conventional MRI signs of soft-tissue tumors.

Parameters	Benign	Intermediate	Malignant	P valuea	Benign vs intermediate	P valueb	Benign vs malignant	P valuec
	(n = 18)	(n = 18)	(n = 19)			Intermediate vs malignant		Benign vs non-benign
Tumor size (cm)	4.31 ± 1.99	5.42 ± 2.17	6.21 ± 2.57	0.043	0.437	0.877	0.040	0.023
Rald	0.54 ± 0.16	0.62 ± 0.17	0.64 ± 0.15	0.178	–	–	–	0.261
T1WI predominant SI				0.311	–	–	–	0.128
Iso-/hypointense	14/18 (77.8)	10/18 (55.6)	11/19 (57.9)					
Hyperintense	4/18 (22.2)	8/18 (44.4)	8/19 (42.1)					
T2WI predominant SI				0.198	–	–	–	0.173
Iso-/hypointense	3/18 (16.7)	2/18 (11.1)	0/19 (0)					
Hyperintense	15/18 (83.3)	16/18 (88.9)	19/19 (100)					
Heterogeneous SI on T1WI				0.604	–	–	–	0.218
≥30% heterogeneity regions	3/18 (16.7)	5/18 (27.8)	7/19(36.8)					
<30% heterogeneity regions	15/18 (83.3)	13/18 (72.2)	12/19 (63.2)					
Heterogeneous SI on T2WI				0.023	0.738	0.009	0.020	0.282
≥30% heterogeneity regions	10/18 (55.6)	9/18 (50.0)	17/19 (89.5)					
<30% heterogeneity regions	8/18 (44.4)	9/18 (50.0)	2/19 (10.5)					
Heterogeneous SI on contrast enhancement T1WI				0.041	0.729	0.029	0.013	0.111
≥30% heterogeneity regions	11/18 (61.1)	12/18 (66.7)	18/19 (94.7)					
<30% heterogeneity regions	7/18 (38.9)	6/18 (33.3)	1/19 (5.3)					
Tissue layer				0.885	–	–	–	0.637
Deep	9/18 (50.0)	8/18 (44.4)	8/19 (42.1)					
Superficial	9/18 (50.0)	10/18 (55.6)	11/19 (57.9)					
Margin				0.169	–	–	–	0.061
Poorly defined	4/18 (22.2)	9/18 (50.0)	9/19 (47.4)					
Well defined	14/18 (77.8)	9/18 (50.0)	10/19 (52.6)					
Shape				0.285	–	–	–	0.283
Lobulated	6/18 (33.3)	7/18 (38.9)	11/19 (57.9)					
Oval	12/18 (66.7)	11/18 (61.1)	8/19 (42.1)					
Band sign				0.629	–	–	–	0.413
Present	14/18 (77.8)	15/18 (83.3)	17/19 (89.5)					
Absent	4/18 (22.2)	3/18 (16.7)	2/19 (10.5)					
Necrosis				<0.001	0.289	0.001	<0.001	0.005
Present	1/18 (5.6)	3/18 (16.7)	11/19 (57.9)					
Absent	17/18 (94.4)	15/18 (83.3)	8/19 (42.1)					
Flow void				0.276	–	–	–	0.127
Present	2/18 (11.1)	6/18 (33.3)	5/19 (26.3)					
Absent	16/18 (88.9)	12/18 (66.7)	14/19 (73.7)					
Tail-like pattern				0.013	0.034	0.585	0.009	0.004
Present	9/18 (50.0)	15/18 (83.3)	17/19 (89.5)					
Absent	9/18 (50.0)	3/18 (16.7)	2/19 (10.5)					
Invasiveness				0.028	0.018	0.772	0.033	0.008
Present	7/18 (38.9)	14/18 (77.8)	14/19 (73.7)					
Absent	11/18 (61.1)	4/18 (22.2)	5/19 (26.3)					
Peritumoral edema				0.091	–	–	–	0.103
Present	6/18 (33.3)	8/18 (44.4)	13/19 (68.4)					
Absent	12/18 (66.7)	10/18 (55.6)	6/19 (31.6)					
Peritumoral enhancement				0.502	–	–	–	0.391
Present	8/18 (44.4)	9/18 (50.0)	12/19 (63.2)					
Absent	10/18 (55.6)	9/18 (50.0)	7/19 (36.8)					

R_ald_, Ratio of axial and lateral diameter; T1WI, T1-weighted image; SI, signal intensity; T2WI, T2-weighted image. ^a^P values were calculated from Kruskal–Wallis H test or chi-squared test.^b^P values were adjusted for pairwise comparison using*post hoc* test with Bonferroni correction.^c^P values were calculated from Mann–Whitney U test or chi-squared test.

### Quantitative diffusion parameters analysis

The mean and minimum values of standard-ADC, D, and f values significantly differed among benign, intermediate, and malignant fibrous STTs (*f_mean_* value:*p* = 0.003,*f_min_* value:*p* = 0.009, other parameters:*p* < 0.001, Kruskal–Wallis H test) (**Table 5**). The malignant tumor group exhibited significantly lower standard-ADC_mean_, standard-ADC_min_, D_mean_, and D_min_ values compared with the benign tumor group (all*p* < 0.001), whereas the f_mean_ and f_min_ values were significantly higher (*p* = 0.002 and 0.013, respectively). Standard-ADC_mean_, standard-ADC_min_, and D_mean_ values were lower in the malignant group than in the intermediate group (*p* = 0.024, 0.013, and 0.005, respectively). Standard-ADC_mean_, D_mean_, and D_min_ values were lower in the intermediate group than in the benign group (*p* = 0.008, 0.024, and 0.040, respectively). The non-benign tumor group exhibited lower standard-ADC_mean_, standard-ADC_min_, D_mean_, and D_min_ values than the benign tumor group (*p* < 0.001 for all), while the f_mean_ value was higher (*p* = 0.009).

**Table 5:** Diffusion parameters of soft-tissue tumors.

Parameters	Benign (n = 18)	Intermediate (n = 18)	Malignant (n = 19)	P valuea	P valueb			P valuec
					Benign vs intermediate	Intermediate vs malignant	Benign vs malignant	Benign vs non-benign
Standard-ADCmean (×10–3 mm2/s)	1.58 (1.53–1.71)	1.39 (1.34–1.49)	1.25 (1.13–1.35)	<0.001	0.008	0.024	<0.001	<0.001
Standard-ADCmin (×10–3 mm2/s)	1.36 (1.20–1.44)	1.20 (1.14–1.32)	1.04 (0.86–1.10)	<0.001	0.130	0.013	<0.001	<0.001
Dmean (×10–3 mm2/s)	1.43 (1.34–1.51)	1.22 (1.15–1.29)	0.91 (0.87–1.09)	<0.001	0.024	0.005	<0.001	<0.001
Dmin (×10–3 mm2/s)	1.19 (1.05–1.24)	0.98 (0.88–1.08)	0.79 (0.71–0.92)	<0.001	0.040	0.089	<0.001	<0.001
D*mean (×10–3 mm2/s)	12.74 (9.56–17.39)	14.80 (10.53–18.38)	16.15 (13.00–18.95)	0.123				0.060
D*min (×10–3 mm2/s)	5.91 (5.01–8.98)	7.11 (4.64–8.86)	8.02 (6.64–12.45)	0.052				0.108
fmean	12.90 (10.97–15.21)	14.20 (12.40–15.94)	16.70 (14.15–21.00)	0.003	0.790	0.074	0.002	0.009
fmin	7.13 (6.30–8.89)	8.35 (6.97–8.69)	9.71 (8.42–11.45)	0.009	0.674	0.052	0.013	0.054

ADC, apparent diffusion coefficient; D, true diffusion coefficient; D^*^, pseudo-diffusion coefficient; f, perfusion fraction. Data are medians with interquartile ranges in parentheses.^a^P values were calculated from Kruskal–Wallis H test.^b^*P* values were adjusted for pairwise comparison using*post hoc* test with Bonferroni correction.^c^P values were calculated from Mann–Whitney U test.

**Figure 2:**
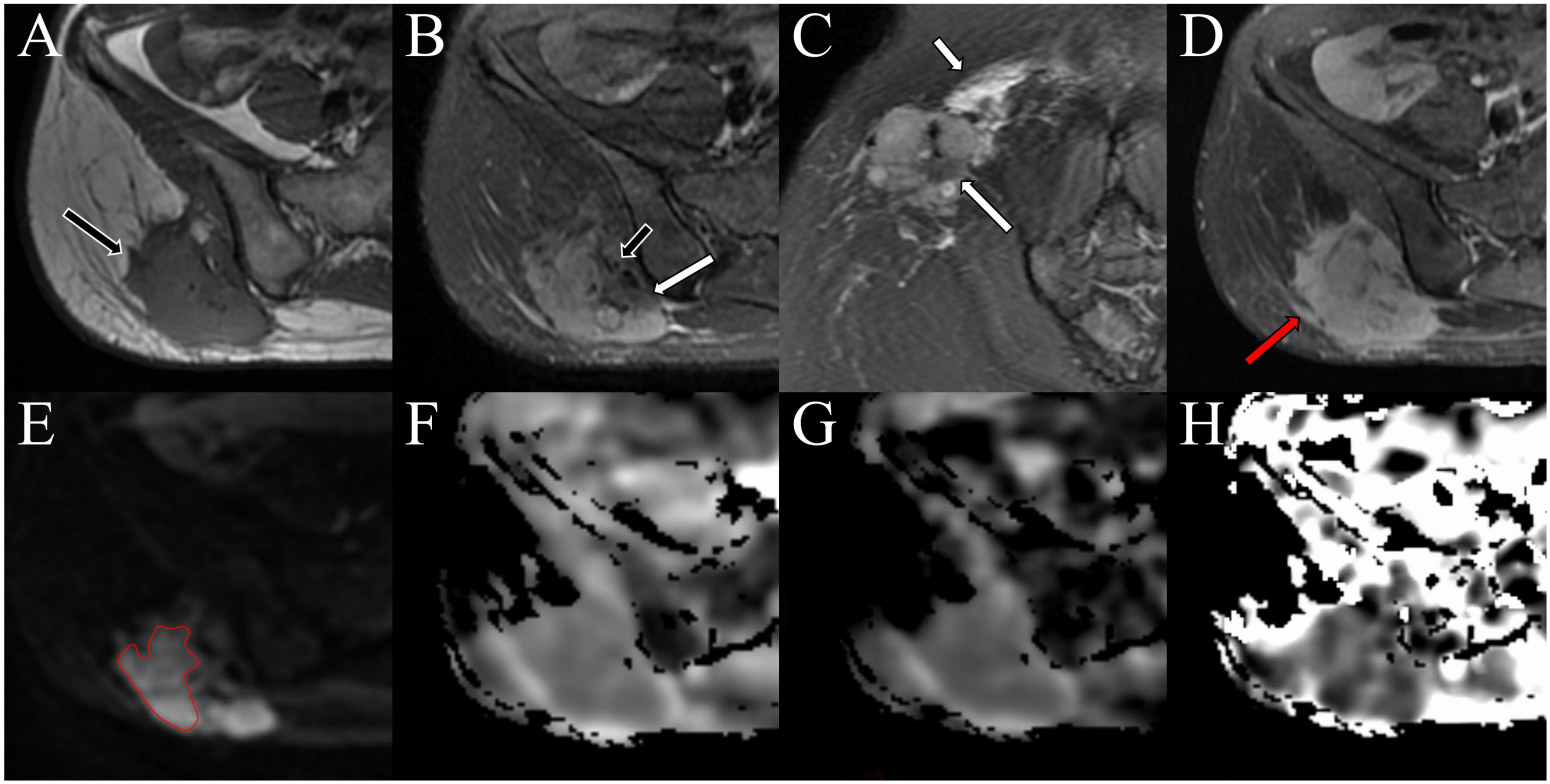
A 39-year-old patient with fibrosarcoma of the right buttock. **(A)** Axial T1-weighted image showed an irregular and slightly hyperintense mass (long black arrow). **(B, C)** Axial and coronal T2-weighted images showed a heterogeneous mass exhibiting internal necrosis, band sign (long white arrow), and flow void (short black arrow), accompanied by peritumoral edema (short white arrow) in the adjacent fat layer. **(D)** Contrast-enhanced T1-weighted image showed heterogeneous enhancement of the lesion, invasion into the surrounding fat space, and presentation of fascial tail sign (long red arrow). **(E)** The solid portion of the lesion showed marked hyperintensity on the IVIM (b = 800 s/mm^2^) image; **(F)** the standard-ADC_mean_ and standard-ADC_min_ values were 1.13 × 10–^3^ mm^2^/s and 1.05 × 10–^3^ mm^2^/s; **(G)** the D_mean_ and D_min_ values were 0.91 × 10^-3^mm^2^/s and 0.72 × 10–^3^ mm^2^/s; and **(H)** the f_mean_ and f_min_ values were 12.90 and 7.81.

**Figure 3:**
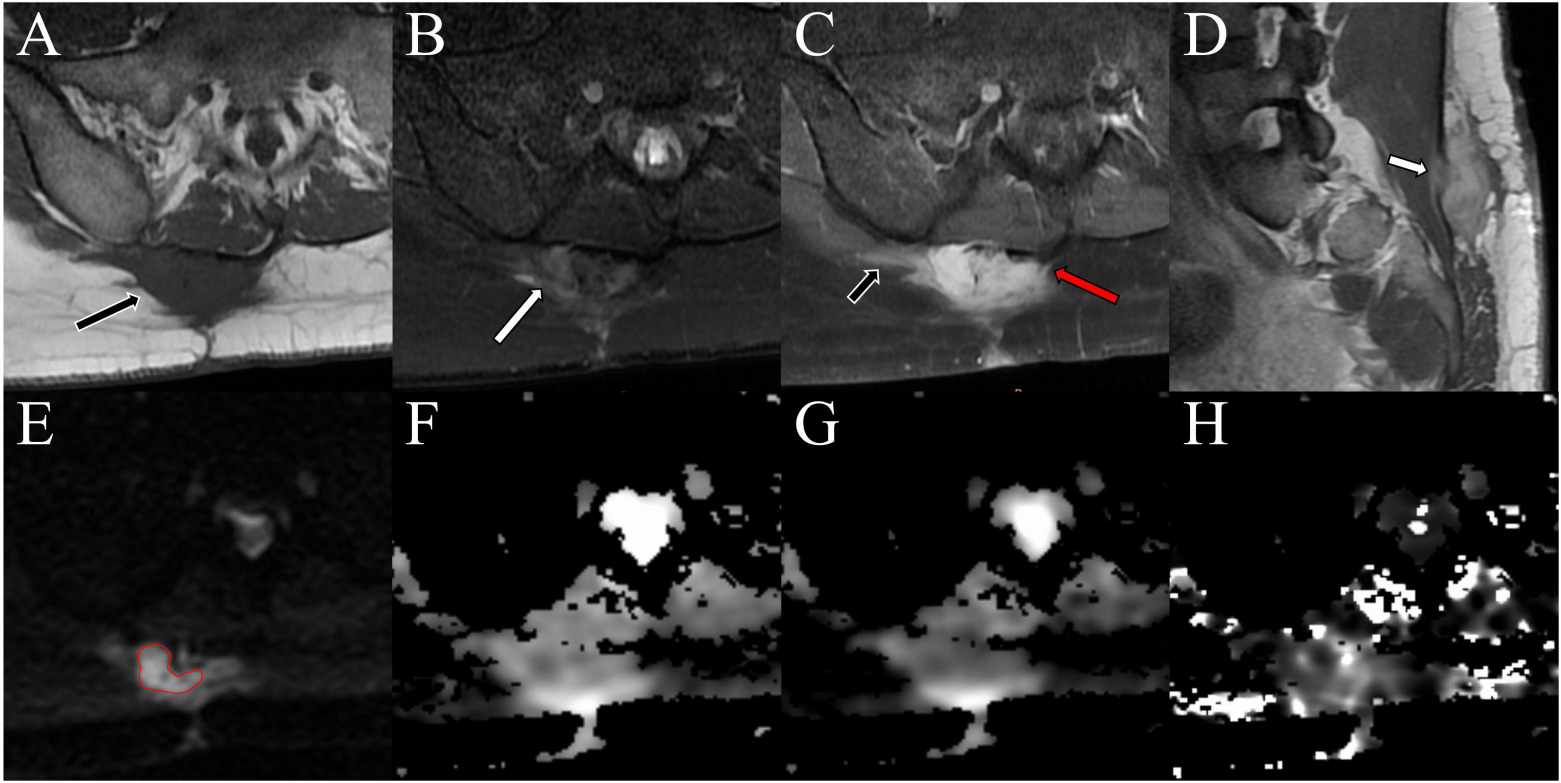
A 32-year-old patient with desmoid-type fibromatosis of the back. **(A)** Axial T1-weighted image showed an irregular hypointense mass (long black arrow). **(B)** Axial T2-weighted images showed a heterogeneous mass with a band sign (long white arrow), extending towards the skin area. **(C, D)** Contrast-enhanced T1-weighted image showed heterogeneous enhancement of the lesion, invasion into the surrounding fat space and erector spinae (short white arrow), and presentation of fascial tail sign (long red arrow) and peritumoral enhancement (short black arrow). **(E)** The solid portion of the lesion showed marked hyperintensity on the IVIM (b = 800 s/mm^2^) image; **(F)** the standard-ADC_mean_ and standard-ADC_min_ values were 1.29 × 10–^3^ mm^2^/s and 1.06 × 10–^3^ mm^2^/s; **(G)** the D_mean_ and D_min_ values were 1.18 × 10–^3^ mm^2^/s and 0.83 × 10–^3^ mm^2^/s; and **(H)** the f_mean_ and f_min_ values were 17.70 and 7.47.

**Figure 4:**
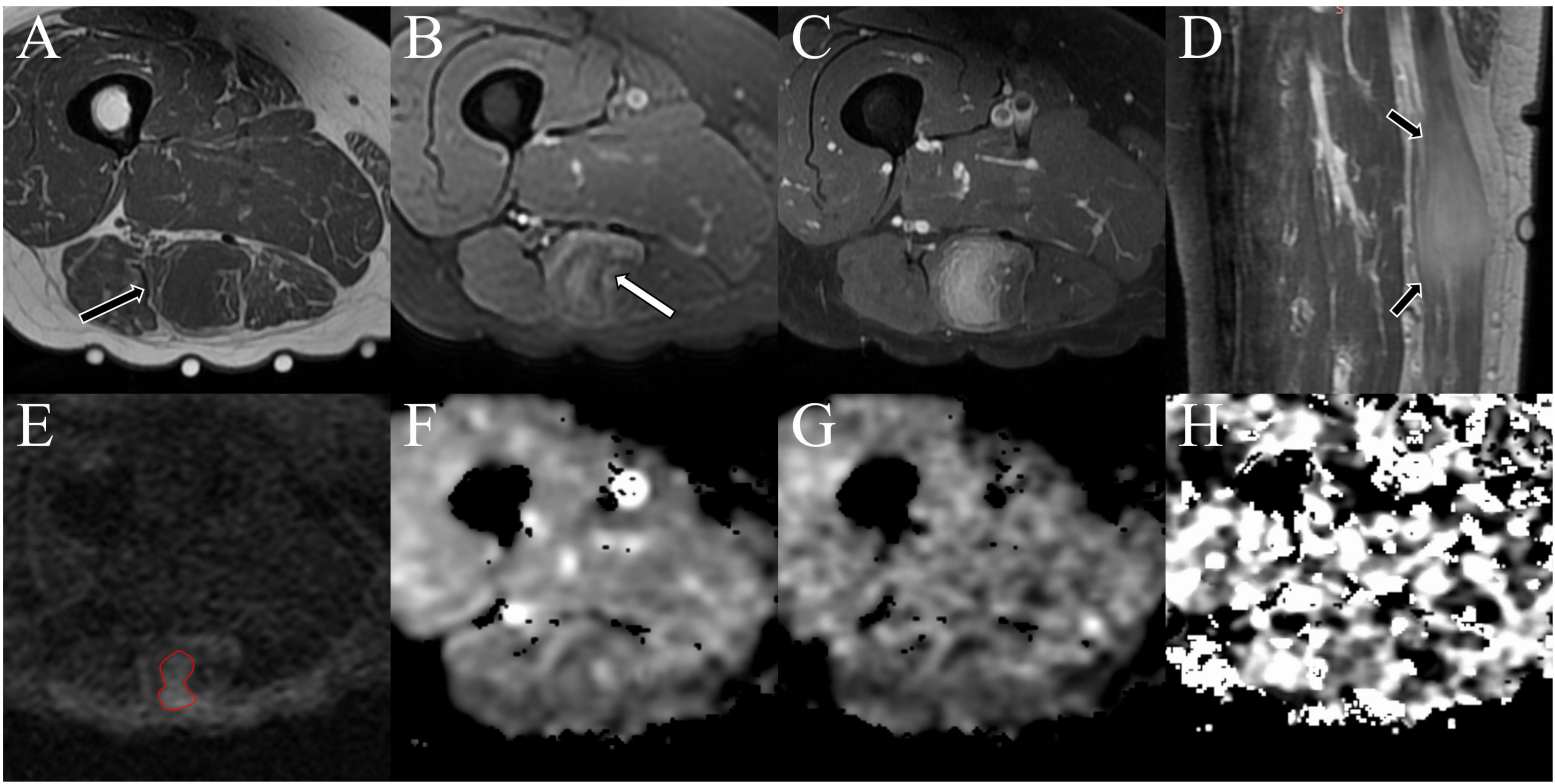
A 76-year-old patient with proliferative myositis of the right thigh.**(A)** Axial T1-weighted image showed a hypointense mass (long black arrow).**(B)** Axial T2-weighted images showed a heterogeneous mass with a band sign (long white arrow).**(C, D)** Contrast-enhanced T1-weighted image showed homogeneous enhancement of the lesion with peritumoral enhancement (short black arrow).**(E)** The solid portion of the lesion showed slightly heterogeneous hyperintense on the IVIM (b = 800 s/mm^2^) image;**(F)** the standard-ADC_mean_ and standard-ADC_min_ values were 1.58 × 10–^3^ mm^2^/s and 1.36 × 10–^3^ mm^2^/s;**(G)** the D_mean_ and D_min_ values were 1.39 × 10–^3^ mm^2^/s and 1.04 × 10–^3^ mm^2^/s; and**(H)** the f_mean_ and f_min_ values were 11.40 and 8.94.

The conventional MRI and diffusion characteristics for histologically confirmed cases of fibrosarcoma, desmoid-type fibromatosis, and proliferative myositis are shown in **Figures 2**–**4**, respectively.

### Diagnostic performance

The diagnostic performance of conventional MRI features in distinguishing between the three STT groups is detailed in**Supplementary Table 2**. For the discrimination of benign*vs*. intermediate fibrous STTs, invasiveness yielded the highest AUC of 0.694, with a sensitivity of 77.8% and a specificity of 61.1%. For the discrimination of benign*vs*. malignant fibrous STTs, necrosis showed the highest AUC of 0.814, with a sensitivity of 68.4% and a specificity of 94.4%. For the discrimination of intermediate*vs*. malignant fibrous STTs, heterogeneous SI on T2WI showed the highest AUC of 0.697, with a sensitivity of 68.4% and a specificity of 83.3%.

**Figure 5:**
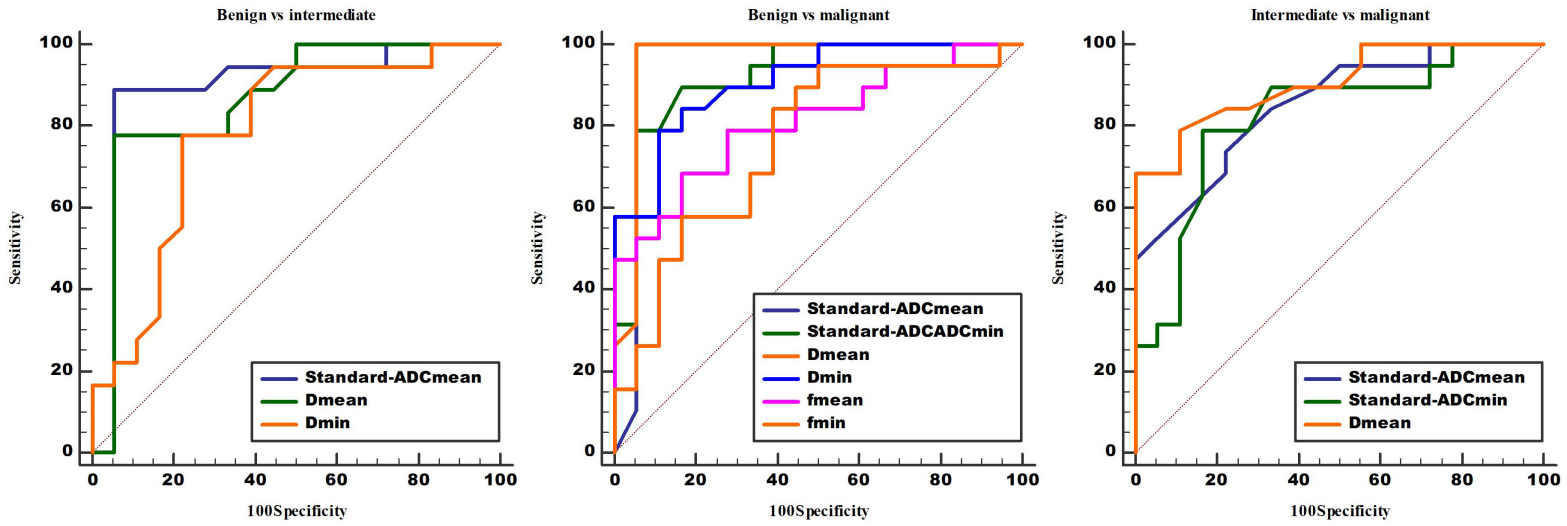
ROC curves showing the diagnostic performance of IVIM parameters for differentiating benign, intermediate, and malignant fibrous STTs.

The diagnostic performance of quantitative diffusion parameters in distinguishing between the three STT groups is detailed in **Table 6** and **Figure 5**. For the discrimination of benign*vs*. intermediate fibrous STTs, standard-ADC_mean_ value yielded the highest AUC of 0.894, with a cutoff value of 1.50, a sensitivity of 88.9%, and a specificity of 94.4%; the AUC for standard-ADC_mean_ value did not significantly differ from those for D_mean_ and D_min_ values (*p* = 0.642 and 0.187, respectively). For the discrimination of benign*vs*. malignant fibrous STTs, D_mean_ value achieved the highest AUC of 0.961, with a cutoff value of 1.25, a sensitivity of 100.0%, and a specificity of 94.4%; the AUC of D_mean_ value was higher than that of f_min_ (0.961*vs*. 0.757, *p* = 0.034), but the AUC of D_mean_ value did not significantly differ from those of standard-ADC_mean_, standard-ADC_min_, D_min_, and f_mean_ values (*p* = 0.354, 0.166, 0.244, and 0.080, respectively). For the discrimination of intermediate*vs*. malignant fibrous STTs, D_mean_ value showed the highest AUC of 0.905, with a cutoff value of 1.01, a sensitivity of 68.4%, and a specificity of 100.0%; the AUC for D_mean_ value did not significantly differ from those for standard-ADC_mean_ and standard-ADC_min_ values (*p* = 0.350 and 0.167, respectively).

**Table 6:** Performance of quantitative diffusion parameters for diagnosing benign, intermediate, and malignant fibrous soft-tissue tumors.

Parameters	AUC	95% CI	Cutoff value	Youden index	Sensitivity (%)	Specificity (%)	Accuracy (%)
Benign vs intermediate							
Standard-ADCmean (×10–3 mm2/s)	0.894	0.745–0.971	≤1.50	0.833	88.9 (16/18)	94.4 (17/18)	91.7 (33/36)
Dmean (×10–3 mm2/s)	0.864	0.709–0.955	≤1.26	0.722	77.8 (14/18)	94.4 (17/18)	86.1 (31/36)
Dmin (×10–3 mm2/s)	0.782	0.614–0.902	≤1.06	0.556	77.8 (14/18)	77.8 (14/18)	77.8 (28/36)
Benign vs malignant							
Standard-ADCmean (×10–3 mm2/s)	0.947	0.820–0.994	≤1.46	0.944	100.0 (19/19)	94.4 (17/18)	97.3 (36/37)
Standard-ADCmin (×10–3 mm2/s)	0.921	0.784–0.984	≤1.10	0.734	79.0 (15/19)	94.4 (17/18)	86.5 (32/37)
Dmean (×10–3 mm2/s)	0.961	0.839–0.997	≤1.25	0.944	100.0 (19/19)	94.4 (17/18)	97.3 (36/37)
Dmin (×10–3 mm2/s)	0.908	0.766–0.978	≤0.92	0.678	79.0 (15/19)	88.9 (16/18)	83.8 (31/37)
fmean	0.810	0.647–0.920	>15.98	0.518	68.4 (13/19)	83.3 (15/18)	75.7 (28/37)
fmin	0.757	0.589–0.883	>7.94	0.453	84.2 (16/19)	61.1 (11/18)	73.0 (27/37)
Intermediate vs malignant							
Standard-ADCmean (×10–3 mm2/s)	0.853	0.697–0.947	≤1.33	0.515	73.7 (14/19)	77.8 (14/18)	75.7 (28/37)
Standard-ADCmin (×10–3 mm2/s)	0.822	0.661–0.928	≤1.10	0.623	79.0 (15/19)	83.3 (15/18)	81.1 (30/37)
Dmean (×10–3 mm2/s)	0.905	0.763–0.976	≤1.01	0.684	68.4 (13/19)	100.0 (18/18)	83.8 (31/37)

AUC, area under the curve; CI, confidence interval; ADC, apparent diffusion coefficient; D, true diffusion coefficient; f, perfusion fraction.

**Figure 6:**
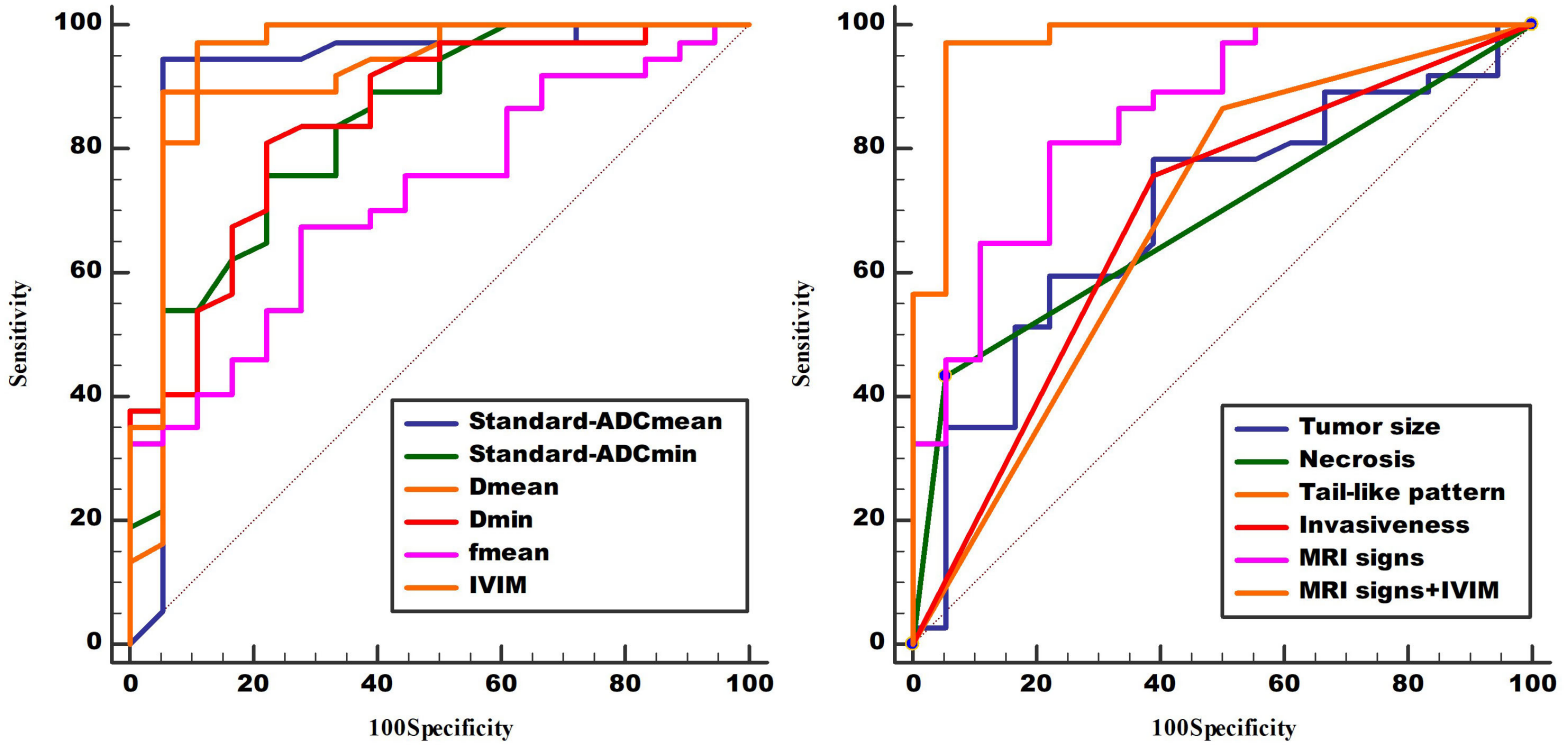
ROC curves showing the diagnostic performance of conventional MRI signs and IVIM parameters for differentiating benign and non-benign fibrous STTs.

For discriminating between benign and non-benign fibrous STTs, standard-ADC_mean_ value showed the highest AUC (0.921) among the single parameters. The combination of conventional MRI signs yielded an AUC of 0.851, a sensitivity of 81.1%, and a specificity of 77.8%. The combination of IVIM parameters yielded an AUC of 0.950, a sensitivity of 97.3%, and a specificity of 88.9%. The combination of conventional MRI signs and IVIM parameters yielded an AUC of 0.971, a sensitivity of 97.3%, and a specificity of 94.4%. The AUC of the combined model was higher than that of the MRI signs (0.971*vs*. 0.851,*p* = 0.038), but did not differ from that of IVIM parameters (*p* = 0.177) (**Table 7**; **Figure 6**).

**Table 7:** Performance of imaging parameters for diagnosing benign and non-benign fibrous soft-tissue tumors.

Parameters	AUC	95% CI	Cutoff value	Youden index	Sensitivity (%)	Specificity (%)	Accuracy (%)
Standard-ADCmean (×10–3 mm2/s)	0.921	0.816–0.977	≤1.50	0.890	94.6 (35/37)	94.4 (17/18)	94.5 (52/55)
Standard-ADCmin (×10–3 mm2/s)	0.836	0.712–0.922	≤1.20	0.535	75.7 (28/37)	77.8 (14/18)	76.3 (42/55)
Dmean (×10–3 mm2/s)	0.914	0.806–0.972	≤1.26	0.836	89.2 (33/37)	94.4 (17/18)	90.9 (50/55)
Dmin (×10–3 mm2/s)	0.847	0.724–0.930	≤1.06	0.589	81.1 (30/37)	77.8 (14/18)	80.0 (44/55)
fmean	0.719	0.582–0.832	>14.05	0.398	67.6 (25/37)	72.2 (13/18)	69.1 (38/55)
Tumor size	0.696	0.577–0.813	>3.8	0.395	78.4 (29/37)	61.1 (11/18)	72.7 (40/55)
Necrosis	0.688	0.549–0.806	/	0.377	43.2 (16/37)	94.4 (17/18)	60.0 (33/55)
Tail-like pattern	0.682	0.543–0.891	/	0.365	86.5 (32/37)	50.0 (9/18)	74.5 (41/55)
Invasiveness	0.684	0.545–0.803	/	0.368	75.7 (28/37)	61.1 (11/18)	70.9 (39/55)
IVIM	0.950	0.856–0.991	/	0.862	97.3 (36/37)	88.9 (16/18)	94.5 (52/55)
MRI signs	0.851	0.730–0.933	/	0.589	81.1 (30/37)	77.8 (14/18)	80.0 (44/55)
MRI signs+IVIM	0.971	0.887–0.998	/	0.917	97.3 (36/37)	94.4 (17/18)	96.3 (53/55)

AUC, area under the curve; CI, confidence interval; ADC, apparent diffusion coefficient; D, true diffusion coefficient; f, perfusion fraction; IVIM, intravoxel incoherent motion.

## Discussion

In this study, we examined the use of MRI features and IVIM parameters for the differential diagnosis of fibrous STTs. Our results showed that conventional MRI features, including tumor size, heterogeneous SI on T2WI, heterogeneous SI on contrast enhancement T1WI, necrosis, tail-like pattern, and invasiveness, and IVIM parameters, such as the standard-ADC_mean_, standard-ADC_min_, D_mean_, and D_min_ values, effectively differentiated benign, intermediate, and malignant fibrous STTs.

In fibroblastic/myofibroblastic tumors, the hyperintense areas on T2WI generally indicate areas of high spindle cell density, while linear hypointense areas (band sign) on T2WI correspond to collagen fibers (7,8). The band sign is a characteristic feature of fibrous STTs and was observed in 83.6% (46/55) of the tumors in our study. However, band sign is not specific and occurs in other STTs such as giant cell tumors of the tendon sheath (9,10). Previous studies identified signal heterogeneity as a characteristic of malignant STTs (21,22), consistent with our findings. Fibrosarcomas exhibit marked cell atypia and active nuclear division, yielding more heterogeneous MR signals relative to benign and intermediate STTs. Our study also revealed that malignant fibrous STTs are more susceptible to necrosis than benign and intermediate STTs, which likely results from rapid tumor growth, inadequate blood supply, and reduced perfusion in the tumor core (23).

We found that tumor size increases with malignancy of fibrous STTs, which is in agreement with previous reports in STTs (24,25). However, contrary to the findings of Gruber et al. (24), we found no significant difference in Rald among the three STT subgroups; this may be attributed to the diverse anatomical origins of the included tumors. Some reports showed that peritumoral edema, peritumoral enhancement, and unclear boundaries are associated with larger malignant tumors as a result of the stimulation of tumor cells on surrounding tissues and high vascular activity (13,25,26). However, our results indicated these signs have limited diagnostic utility for differentiating fibrous tumors. Additionally, the depth of fibrous STTs showed no correlation with the degree of malignancy in this study, which is consistent with previous findings (25).

Invasiveness is not unique to malignancies, while some intermediate tumors exhibit local aggressiveness, such as SFTs and DFs, and the latter often infiltrates surrounding tissues in a root-like pattern, leading to misdiagnosis of malignant tumors (7,8). We found that invasive signs were more common in the malignant and intermediate groups, which is consistent with their biological characteristics. The tail-like pattern is associated with higher local recurrence rates and poorer prognosis (27,28). In this study, malignant and intermediate lesions more frequently exhibited this tail-like linear extension. The accurate identification of invasiveness and a tail-like pattern is essential for achieving negative surgical margins. Kaga et al. reported that a flow void was observed more frequently in SFT than in DF (8). In our study, the flow void did not differ significantly among the three fibrous STT groups.

Standard-ADC and D values both reflect restricted water diffusion in tissues and are primarily influenced by cell density. Consistent with earlier reports (17–19), the standard-ADC_mean_, standard-ADC_min_, D_mean_, and D_min_ values gradually declined from benign to non-benign (intermediate and malignant) fibrous STTs. Standard-ADC_mean_, D_mean_, and D_min_ values were found to distinguish benign from intermediate and malignant tumors. Standard-ADC_mean_, standard-ADC_min_, and D_mean_ values distinguished intermediate from malignant lesions. As the malignancy of the tumor increases, tumor cells proliferate more and are more densely distributed, which reduces the extracellular space and thus lowers the standard-ADC and D values. The ADC_min_ reflects regions of maximal cellularity and restricted diffusion, reportedly enabling more precise identification of malignant lesions than ADC_mean_ (29,30). However, we found that standard-ADC_mean_ and D_mean_ values showed higher diagnostic performance than standard-ADC_min_ and D_min_ values in differentiating benign from non-benign fibrous STTs, which is consistent with previous findings (31).

The stability and repeatability of IVIM-derived parameters are affected by b-value combinations, especially the perfusion-related D^*^ and f values. Previous study has reported that incorporating more b-values below 200 s/mm^2^ (the perfusion-sensitive range) improves the measurement reliability of D^*^ and f values (32). Poor inter-observer agreement of the f value was observed in our study, which was consistent with an earlier study focusing on STTs (25). The D^*^ values failed to differentiate STTs in the present work, probably owing to the relatively poor vascularization of fibrous tumors. Although significantly elevated f_mean_ and f_min_ values were found in malignant STTs compared with benign counterparts, their diagnostic performance was inferior to other quantitative parameters. These findings indicate that D^*^ and f values may not serve as reliable indicators for the preoperative differentiation of fibrous STTs.

ROC analysis revealed that D value outperformed conventional MRI features for subgroup discrimination of tumors. The combined model integrating conventional MRI findings and IVIM parameters yielded the optimal diagnostic performance to separate benign from non-benign lesions. On this basis, D value could further stratify intermediate and malignant tumors within the non-benign cohort. Accurate preoperative characterization enables timely personalized therapy, and this multiparametric MRI combination provides robust imaging evidence to refine clinical decision-making and stratified management for patients with fibrous STTs. Progressive malignant transformation complicates tissue microstructure, restricting the diagnostic utility of diffusion parameters. Future research combining advanced diffusion models, such as the stretched-exponential model and diffusion kurtosis imaging model, to more accurately reflect the diffusion, perfusion, and heterogeneity of STTs is required.

This study has several limitations. First, limited overall sample size and imbalanced subgroup distribution raise concerns regarding overfitting of high AUC values. In addition, cases with abundant myxoid stroma were excluded due to their propensity to increase ADC measurements (33), which may exacerbate selection bias. Second, there is currently no consensus on the optimal b-value combination for IVIM in STTs. Third, Tumor heterogeneity may lead to ROI measurement errors, as we manually selected the ROI in solid tumor portions rather than the entire tumor. Fourth, there is a lack of detailed research linking imaging findings with clinical pathological information and prognosis of patients, which hindered a more comprehensive evaluation of the implications of the characteristics of fibrous tumors. Therefore, our study only provides preliminary exploratory results. Future multicenter studies with expanded fibrous STT cohorts should refine b-value settings and adopt artificial intelligence analysis for precise assessment of tumor microstructural changes.

## Conclusion

In summary, our results demonstrate that fibrous STTs exhibit diverse conventional MRI signals and morphologies. Invasive signs and tail-like pattern occur more frequently in malignant and intermediate tumors. Malignant tumors also demonstrated greater signal heterogeneity and a higher incidence of necrosis. IVIM parameters can provide a quantitative basis for the differential diagnosis of fibrous STTs. Both the mean and minimum values of standard-ADC and D gradually decrease as the degree of malignancy increases. The combination of conventional MRI signs and IVIM parameters allows specific and non-invasive discrimination of benign from non-benign (intermediate/malignant) fibrous STTs.

## Data Availability

The raw data supporting the conclusions of this article will be made available by the authors, without undue reservation.

## References

[1] Choi JH Ro JY . The 2020 WHO classification of tumors of soft tissue: selected changes and new entities.Adv Anat Pathol. (2021)28:44–58. doi: 10.1097/PAP.0000000000000284 32960834

[2] Ng E Tandon AA Ho BC Chong BK . Characterising benign fibrous soft-tissue tumours in adults: why is it so difficult and what do we need to know?Clin Radiol. (2015)70:684–97. doi: 10.1016/j.crad.2015.02.010 25782339

[3] Riedel RF Agulnik M . Evolving strategies for management of desmoid tumor.Cancer. (2022)128:3027–40. doi: 10.1002/cncr.34332 35670122 PMC9546183

[4] Zhao ZY Chen ZY Yu B Xiao B Liu LY Xia Y . Characterization of the immune cell infiltration landscape in myxofibrosarcoma to aid immunotherapy.Front Immunol. (2022)13:916915. doi: 10.3389/fimmu.2022.916915 35936000 PMC9353264

[5] Gamboa AC Gronchi A Cardona K . Soft-tissue sarcoma in adults: an update on the current state of histiotype-specific management in an era of personalized medicine.CA Cancer J Clin. (2020)70:200–29. doi: 10.3322/caac.21605 32275330

[6] Coyle J White LM Dickson B Ferguson P Wunder J Naraghi A . MRI characteristics of nodular fasciitis of the musculoskeletal system.Skeletal Radiol. (2013)42:975–82. doi: 10.1007/s00256-013-1620-9 23624727

[7] Tanishima T Kurokawa R Sone M Kusumoto M Abe O . Radiological features of desmoid-type fibromatosis: a two-institution retrospective study.Eur Radiol. (2025)35:1394–404. doi: 10.1007/s00330-024-11285-3 39888408

[8] Kaga T Kato H Kawaguchi M Kanayama T Nagano A Omata S . MRI characteristics for distinguishing solitary fibrous tumor from desmoid tumor.Korean J Radiol. (2025)26:169–79. doi: 10.3348/kjr.2024.0885 39898397 PMC11794291

[9] Finkelstein D Foremny G Singer A Clifford P Pretell-Mazzini J Kerr DA . Differential diagnosis of T2 hypointense masses in musculoskeletal MRI.Skeletal Radiol. (2021)50:1981–94. doi: 10.1007/s00256-021-03711-0 33651128

[10] Ge Y Guo G You Y Li Y Xuan Y Jin ZW . Magnetic resonance imaging features of fibromas and giant cell tumors of the tendon sheath: differential diagnosis.Eur Radiol. (2019)29:3441–9. doi: 10.1007/s00330-019-06226-4 31041564

[11] Wang H Nie P Dong C Li J Huang Y Hao D . CT and MRI findings of soft tissue adult fibrosarcoma in extremities.BioMed Res Int. (2018)2018:6075705. doi: 10.1155/2018/6075705 29693010 PMC5859867

[12] Wang Q Xiao X Liang Y Wen H Wen X Gu M . Diagnostic performance of diffusion MRI for differentiating benign and Malignant nonfatty musculoskeletal soft tissue tumors: a systematic review and meta-analysis.J Cancer. (2021)12:7399–412. doi: 10.7150/jca.62131 35003360 PMC8734420

[13] Choi YJ Lee IS Song YS Kim JI Choi KU Song JW . Diagnostic performance of diffusion-weighted (DWI) and dynamic contrast-enhanced (DCE) MRI for the differentiation of benign from Malignant soft-tissue tumors.J Magn Reson Imaging. (2019)50:798–809. doi: 10.1002/jmri.26607 30663160

[14] Gowda P Bajaj G Silva FD Ashikyan O Xi Y Chhabra A . Does the apparent diffusion coefficient from diffusion-weighted MRI imaging aid in the characterization of Malignant soft tissue tumors and sarcomas.Skeletal Radiol. (2023)52:1475–84. doi: 10.1007/s00256-023-04289-5 36725723

[15] Kwee RM Kwee TC . Diagnostic performance of MRI in detecting locally recurrent soft tissue sarcoma: systematic review and meta-analysis.Eur Radiol. (2022)32:3915–30. doi: 10.1007/s00330-021-08457-w 35020015

[16] Le Bihan D Breton E Lallemand D Aubin ML Vignaud J Laval-Jeantet M . Separation of diffusion and perfusion in intravoxel incoherent motion MR imaging.Radiology. (1988)168:497–505. doi: 10.1148/radiology.168.2.3393671 3393671

[17] Arslan S Ergen FB Aydın GB Ayvaz M Karakaya J Kösemehmetoğlu K . Different attenuation models of diffusion-weighted MR imaging for the differentiation of benign and Malignant musculoskeletal tumors.J Magn Reson Imaging. (2022)55:594–607. doi: 10.1002/jmri.27887 34399016

[18] Lee SK Jee WH Jung CK Chung YG . Multiparametric quantitative analysis of tumor perfusion and diffusion with 3T MRI: differentiation between benign and Malignant soft tissue tumors.Br J Radiol. (2020)93:20191035. doi: 10.1259/bjr.20191035 32649224 PMC8519635

[19] Wu H Zhang S Liang C Liu H Liu Y Mei Y . Intravoxel incoherent motion MRI for the differentiation of benign, intermediate, and Malignant solid soft-tissue tumors.J Magn Reson Imaging. (2017)46:1611–8. doi: 10.1002/jmri.25733 28419705

[20] Lim HK Jee WH Jung JY Paek MY Kim I Jung CK . Intravoxel incoherent motion diffusion-weighted MR imaging for differentiation of benign and Malignant musculoskeletal tumours at 3 T.Br J Radiol. (2018)91:20170636. doi: 10.1259/bjr.20170636 29144153 PMC5965794

[21] Chung WJ Chung HW Shin MJ Lee SH Lee MH Lee JS . MRI to differentiate benign from Malignant soft-tissue tumours of the extremities: a simplified systematic imaging approach using depth, size and heterogeneity of signal intensity.Br J Radiol. (2012)85:e831–836. doi: 10.1259/bjr/27487871 22553293 PMC3474004

[22] Crombe A Alberti N Stoeckle E Brouste V Buy X Coindre JM . Soft tissue masses with myxoid stroma: can conventional magnetic resonance imaging differentiate benign from Malignant tumors?Eur J Radiol. (2016)85:1875–82. doi: 10.1016/j.ejrad.2016.08.015 27666630

[23] Haseli S Mansoori B Christensen D Abadi A Pooyan A Shomal Zadeh F . Fibroblastic and myofibroblastic soft-tissue tumors: imaging spectrum and radiologic-pathologic correlation.Radiographics. (2023)43:e230005. doi: 10.1148/rg.230005 37440448

[24] Gruber L Loizides A Ostermann L Glodny B Plaikner M Gruber H . Does size reliably predict Malignancy in soft tissue tumours?Eur Radiol. (2016)26:4640–8. doi: 10.1007/s00330-016-4300-z 26960540

[25] Zhang K Dai Y Yu C Liu J Cheng Y Zhou Y . Differentiation of benign, intermediate, and Malignant soft-tissue tumours by using multiple diffusion-weighted imaging models.Clin Radiol. (2025)86:106942. doi: 10.1016/j.crad.2025.106942 40403342

[26] Jin K Yoon MA Kim DY . Small (≤5 cm) soft tissue tumors of the extremity and superficial trunk: MRI features and demographic characteristics associated with Malignancy.Acta Radiol. (2023)64:1886–95. doi: 10.1177/02841851221143656 36471487

[27] Yoo HJ Hong SH Kang Y Choi JY Moon KC Kim HS . MR imaging of myxofibrosarcoma and undifferentiated sarcoma with emphasis on tail sign; diagnostic and prognostic value.Eur Radiol. (2014)24:1749–57. doi: 10.1007/s00330-014-3181-2 24889995

[28] Kikuta K Kubota D Yoshida A Morioka H Toyama Y Chuuman H . An analysis of factors related to the tail-like pattern of myxofibrosarcoma seen on MRI.Skeletal Radiol. (2015)44:55–62. doi: 10.1007/s00256-014-1992-5 25172220

[29] Kang Y Choi SH Kim YJ Kim KG Sohn CH Kim JH . Gliomas: histogram analysis of apparent diffusion coefficient maps with standard- or high-b-value diffusion-weighted MR imaging--correlation with tumor grade.Radiology. (2011)261:882–90. doi: 10.1148/radiol.11110686 21969667

[30] Kato F Kudo K Yamashita H Wang J Hosoda M Hatanaka KC . Differences in morphological features and minimum apparent diffusion coefficient values among breast cancer subtypes using 3-tesla MRI.Eur J Radiol. (2016)85:96–102. doi: 10.1016/j.ejrad.2015.10.018 26724653

[31] Song Y Yoon YC Chong Y Seo SW Choi YL Sohn I . Diagnostic performance of conventional MRI parameters and apparent diffusion coefficient values in differentiating between benign and Malignant soft-tissue tumours.Clin Radiol. (2017)72:691.e1–691.e10. doi: 10.1016/j.crad.2017.02.003 28274509

[32] Koh DM Collins DJ Orton MR . Intravoxel incoherent motion in body diffusion-weighted MRI: reality and challenges.AJR Am J Roentgenol. (2011)196:1351–61. doi: 10.2214/AJR.10.5515 21606299

[33] Maeda M Matsumine A Kato H Kusuzaki K Maier SE Uchida A . Soft-tissue tumors evaluated by line-scan diffusion-weighted imaging: influence of myxoid matrix on the apparent diffusion coefficient.J Magn Reson Imaging. (2007)25:1199–204. doi: 10.1002/jmri.20931 17520732

