# Supplementary material for: Intravoxel incoherent motion combined with conventional MRI for the differentiation of benign, intermediate, and malignant fibrous soft-tissue tumors
Source: Front Oncol. 2026 Jun 26;16:1863609. doi: 10.3389/fonc.2026.1863609 (PMC13349916; doi:10.3389/fonc.2026.1863609)
Supplement: Supplementary file 1 [file Table1.docx]

**Supplementary Table 1** ICC analysis of quantitative parameters stratified by lesion location

| Parameters | Trunk | Upper limb | Thigh | Shoulder | Distal lower limb | Hip |
| --- | --- | --- | --- | --- | --- | --- |
| Standard-ADC_mean_  (×10^-3^ mm^2^/s) | 0.868 (0.653–0.954) | 0.815 (0.478–0.943) | 0.974 (0.890–0.994) | 0.687 (-0.039–0.938) | 0.869 (0.334–0.981) | 0.968 (0.793–0.995) |
| Standard-ADC_min_  (×10^-3^ mm^2^/s) | 0.786 (0.473-0.923) | 0.630 (0.118–0.877) | 0.945 (0.778–0.987) | 0.896 (0.515–0.981) | 0.958 (0.733–0.994) | 0.959 (0.741–0.994) |
| D_mean_ (×10^-3^ mm^2^/s) | 0.936 (0.820-0.978) | 0.854 (0.848–0.986) | 0.898 (0.615–0.976) | 0.815 (0.225–0.966) | 0.979 (0.862–0.997) | 0.959 (0.738–0.994) |
| D_min_ (×10^-3^ mm^2^/s) | 0.684 (0.283-0.881) | 0.969 (0.896–0.991) | 0.883 (0.567–0.972) | 0.948 (0.733–0.991) | 0.960 (0.747–0.994) | 0.760 (0.014–0.963) |
| D^*^_mean_ (×10^-3^ mm^2^/s) | 0.843 (0.596-0.978) | 0.525 (-0.040–0.835) | 0.952 (0.805–0.989) | 0.743 (0.077–0.951) | 0.982 (0.878–0.997) | 0.484 (-0.426–0.907) |
| D^*^_min_ (×10^-3^ mm^2^/s) | 0.867 (0.650-0.953) | 0.675 (0.194–0.894) | 0.711 (0.270–0.943) | 0.150 (-0.623–0.775) | 0.922 (0.551–0.989) | -0.453 (-0.900–0.458) |
| f_mean_ | 0.569 (0.100-0.831) | 0.529 (-0.034–0.837) | 0.848 (0.465–0.964) | 0.559 (-0.187–0.917) | 0.500 (-0.408–0.911) | 0.504 (-0.404–0.912) |
| f_min_ | 0.265 (-0.268-0.673) | 0.471 (-0.111–0.812) | 0.777 (0.284–0.945) | 0.050 (-0.645–0.726) | 0.765 (0.026–0.963) | 0.300 (-0.588–0.860) |

ICC, Intraclass correlation coefficient; ADC, apparent diffusion coefficient; D, true diffusion coefficient; D^*^, pseudo-diffusion coefficient; f, perfusion fraction.

**Supplementary Table 2** Performance of conventional MRI features for diagnosing benign, intermediate, and malignant fibrous soft-tissue tumors

| Parameters | AUC | 95%CI | Youden index | Sensitivity (%) | Specificity (%) | Accuracy (%) |
| --- | --- | --- | --- | --- | --- | --- |
| **Benign vs intermediate** |  |  |  |  |  |  |
| Tail-like pattern | 0.667 | 0.490–0.814 | 0.333 | 83.3 (15/18) | 50.0 (9/18) | 66.7 (24/36) |
| Invasiveness | 0.694 | 0.519–0.837 | 0.389 | 77.8 (14/18) | 61.1 (11/18) | 69.4 (25/36) |
| **Benign vs malignant** |  |  |  |  |  |  |
| Tumor size | 0.724 | 0.552–0.857 | 0.465 | 63.2 (12/19) | 83.3 (15/18) | 73.0 (27/37) |
| Heterogeneous SI on T2WI | 0.670 | 0.496–0.815 | 0.339 | 89.5 (17/19) | 44.4 (8/18) | 67.6 (25/37) |
| Heterogeneous SI on contrast enhancement T1WI | 0.668 | 0.494–0.814 | 0.336 | 94.7 (18/19) | 38.9 (7/18) | 67.6 (25/37) |
| Necrosis | 0.814 | 0.652–0.923 | 0.629 | 68.4 (13/19) | 94.4 (17/18) | 78.3 (29/37) |
| Tail-like pattern | 0.697 | 0.525–0.837 | 0.395 | 89.5 (17/19) | 50.0 (9/18) | 70.3 (26/37) |
| Invasiveness | 0.674 | 0.500–0.818 | 0.348 | 73.7 (14/19) | 61.1 (11/18) | 67.6 (25/37) |
| **Intermediate vs malignant** |  |  |  |  |  |  |
| Heterogeneous SI on T2WI | 0.697 | 0.525–0.837 | 0.395 | 89.5 (17/19) | 50.0 (9/18) | 70.3 (26/37) |
| Heterogeneous SI on contrast enhancement T1WI | 0.640 | 0.466–0.791 | 0.281 | 94.7 (18/19) | 33.3 (6/18) | 64.9 (24/37) |
| Necrosis | 0.759 | 0.590–0.884 | 0.518 | 68.4 (13/19) | 83.3 (15/18) | 75.7 (28/37) |

AUC, area under the curve; CI, confidence interval; SI, signal intensity; T2WI, T2-weighted image; T1WI, T1-weighted image.
